# Green Chemistry Synthesis of Silver Nanoparticles and Their Potential Anticancer Effects

**DOI:** 10.3390/cancers12040855

**Published:** 2020-04-01

**Authors:** Zubair Ahmed Ratan, Mohammad Faisal Haidere, Md. Nurunnabi, Sadi Md. Shahriar, A.J. Saleh Ahammad, Youn Young Shim, Martin J.T. Reaney, Jae Youl Cho

**Affiliations:** 1 Department of Integrative Biotechnology, Biomedical Institute for Convergence at SKKU (BICS), Sungkyunkwan University, Suwon 16419, Korea; zubairahmed@bme.kuet.ac.bd (Z.A.R.); younyoung.shim@usask.ca (Y.Y.S.); 2 Department of Biomedical Engineering, Khulna University of Engineering and Technology, Khulna 9203, Bangladesh; 3 Department of Public Health and Informatics, Bangabandhu Sheikh Mujib Medical University, Dhaka 1000, Bangladesh; mfaisal.dhaka@gmail.com; 4 Department of Pharmaceutical Sciences, School of Pharmacy, University of Texas at El Paso, El Paso, TX 79902, USA; mnurunnabi@utep.edu; 5 Department of Materials Science and Engineering, Khulna University of Engineering and Technology, Khulna 9203, Bangladesh; sadimdshahriar@mse.kuet.ac.bd; 6 Department of Chemistry, Jagannath University, Dhaka 1100, Bangladesh; ajsahammad@chem.jnu.ac.bd; 7 Department of Plant Sciences, University of Saskatchewan, Saskatoon, SK S7N 5A8, Canada; 8 Guangdong Saskatchewan Oilseed Joint Laboratory, Department of Food Science and Engineering, Jinan University, Guangzhou 510632, China

**Keywords:** silver nanoparticles, green chemistry, cancer, anti-cancer effect

## Abstract

Nanobiotechnology has grown rapidly and become an integral part of modern disease diagnosis and treatment. Biosynthesized silver nanoparticles (AgNPs) are a class of eco-friendly, cost-effective and biocompatible agents that have attracted attention for their possible biomedical and bioengineering applications. Like many other inorganic and organic nanoparticles, such as AuNPs, iron oxide and quantum dots, AgNPs have also been widely studied as components of advanced anticancer agents in order to better manage cancer in the clinic. AgNPs are typically produced by the action of reducing reagents on silver ions. In addition to numerous laboratory-based methods for reduction of silver ions, living organisms and natural products can be effective and superior source for synthesis of AgNPs precursors. Currently, plants, bacteria and fungi can afford biogenic AgNPs precursors with diverse geometries and surface properties. In this review, we summarized the recent progress and achievements in biogenic AgNPs synthesis and their potential uses as anticancer agents.

## 1. Introduction

Cancer is one of the leading causes of mortality, resulting in one in six deaths (or 9.6 million people) in 2018 alone; however, 70% of deaths from cancer occur in middle- and low-income countries [1]. Surgery, chemotherapy, radiation therapy and hormone therapy are the most common approaches for cancer treatment and management. In recent years, therapeutic and diagnostic approaches based on nanotechnology have shown potential to improve cancer therapy [2]. Cancer nanotechnology unfolded a newer horizon of interdisciplinary research across chemistry, medicine, engineering and biology focusing on the major advancement in cancer detection, diagnosis and treatment [3]. In recent years, nanoparticles (NPs) have gained an immense scientific attraction due to their large surface area to volume ratio and high reactivity with unmatchable properties [4]. Recently, nanotechnology-based anticancer drugs, such as Abraxane^®^ (Celgene, Summit, NJ, USA), Doxil^®^ (Johnson & Johnson, New Brunswick, NJ, USA) and Myocet^™^ (Perrigo, Dublin, Ireland), have been approved by the US Food and Drug Administration for clinical use [5].

Particles with at least one dimension smaller than 100 nm are defined as NPs. Their physical (e.g., plasmon resonance and fluorescent enhancement) and chemical (e.g., catalytic activity enhancement) properties arise from their surface geometry, which determines the area/volume ratio. When NPs diameter decreases for a spherical particle, the surface area increases proportionately to the square of diameter and there is a resulting increase of surface activity compared to bulk materials with larger dimensions. In some cases, a decrease in size combined with an increase in the area can improve material biocompatibility [6]. Although nanotechnology is one of the most promising research fields and is currently viewed as a frontier in medicine, only a few nanoproducts are currently considered for bio-application due to their potential toxicity and unknown safety. Silver, a precious metal, has been widely used in medicine since ancient times. Hippocrates, the father of modern medicine, believed that silver could treat disease and promote wound healing [7]. It has fascinating material properties, which are cost-effective as well as abundant in nature compared to other precious metals. Previous studies revealed that the physical, optical and catalytic properties of silver nanoparticles (AgNPs) are strongly influenced by their size, distribution, morphologic shape and surface properties [8]. Hereafter, AgNPs have received considerable attention from scientists for their unique properties [9].

Metallic NPs such as silver, gold and platinum NPs have been widely tested in humans. For medicinal applications, NPs synthesis must be biocompatible and either non-toxic or low-toxicity protocols should be used. The most common method which has been used to produce AgNPs is chemical synthesis, recruiting reagents whose function is to reduce the silver ions and stabilize the nanoparticles. However, these reagents are toxic and may have potential health hazards [10]. In addition, these production methods are usually expensive and labor-intensive. To address this issue, approaches for AgNPs synthesis through green chemistry (such as the use of biologic sources) have shown great promise recently. During the past decade, it has been demonstrated that many biologic systems, including plants, algae, bacteria, yeast and fungi can transform inorganic metal ions into metal nanoparticles via the reductive capacities of the proteins and metabolites present in these organisms. Biologic methods of AgNPs synthesis have been reported using plants [11,12], bacteria [13], fungi [14,15], seaweed [16], algae [17] and lichen [18]. These eco-friendly methods can present alternatives methods to chemical and physical syntheses of AgNPs. Physicochemical properties of AgNPs can have a significant impact on their biologic activity, therefore particle characterization is normally completed after synthesis [19]. In addition, AgNPs synthesized using biologic approaches typically retain a homogenous chemical composition with few defects [20]. These AgNPs show promising activity against a range of cancer cell lines. Such eco-friendly methods provide alternatives to the traditional chemical and physical synthesis of AgNPs for use in anti-cancer treatments.

AgNPs can cause cytotoxicity of the cancer cells by altering their morphology and reducing the viability along with oxidative stress in different cancer cells [21,22]. In addition, AgNPs play a pivotal role in tumor control via their cytotoxic effects [23]. The purpose of this review is to provide an overview of the main published studies concerning the use of biologic synthesis of silver nanoparticles and the applications of these materials in different cancer cell lines. Several biologic synthesis mechanisms of AgNPs are discussed together with the importance of biologically synthesized AgNPs on cancer cell lines.

## 2. Properties of AgNPs

Unique physicochemical properties of AgNPs provides them the ease of entrance into the cells and gives them the ability to efficiently interact with biomolecules inside the cells or on the cell surfaces [24]. Some of the crucial properties of the AgNPs are described below.

### 2.1. Shape and Size

While picking a drug material for the treatment of certain disease types including cancer, hemocompatibility of that material should be kept in mind [25]. Kwon et al. demonstrated the minimization of the damage to membrane including hemolysis, potassium efflux, protein leakage and alterations in cell shape and membrane fragility by the use of AgNPs of specific shape and size [26]. Therefore, AgNPs have been evidenced to have great potential in anti-cancer activity since they show selective participation in the interruption of mitochondrial respiratory chain that leads to the production of reactive oxygen species (ROS) and disruption of adenosine triphosphate (ATP) synthesis, thus resulting in nucleic acid damage [27]. Various studies have established that temperature, pH of the solution, precursor concentration, the molar ratio of capping agent to that of precursor, the types of reducing agents, the strength of chemical interaction between the precursor and different crystallographic planes of AgNPs and the synthesis method determine the size and shape of the AgNPs [28]. Depending on the synthesis methods, a range of particle sizes and shapes (e.g., cubes, prisms, spheres, rods, wires, plates, etc.) can be obtained [29]. For instance, in the synthesis of AgNPs from the bacteria *Xanthomonas oryzae*, spherical as well as triangular and rod-shaped particles with an average size of 14.86 nm were obtained depending on the experimental conditions [30], whereas bacterial synthesis from *Pseudomonas stutzeri* AG259 resulted in triangular and hexagonal particles with an average size of 200 nm [31]. In the fungal synthesis from *Fusarium acuminatum*, spherical AgNPs in the range of 5–40 nm (average size 13 nm) were reported [32]. On the other hand, AgNPs synthesis from the fungus *Trichoderma viride*, spherical and sporadically rod-like particles in the range of 10–40 nm at 27 °C. Decreasing the temperature to 10 °C increased the particle size to 80–100 nm [15]. Studies have established that low reaction temperatures can facilitate the formation of two-dimensional nanostructures [33]. To underscore the range of sizes and shapes of AgNPs which plant-based green synthesis can yield, the study on *Nelumbo nucifera* can be used as an example. Synthesis using the leaf extract of this plant resulted in AgNPs with spherical, triangular, truncated triangular and decahedral morphologies. Sizes of the particles ranged between 25 and 80 mm averaging 45 mm [34]. Vilchis-Nestor et al. reported more spherical and larger AgNPs under ambient conditions with the increase in the concentration of the *Camellia sinensis* (green tea) extracts [35].

### 2.2. Optical Properties

AgNPs efficiently interact with light through a phenomenon known as surface plasmon resonance (SPR) more efficiently than any known organic or inorganic chromophores. This strong interaction is a result of both the restriction of a large density of conducting electrons to smaller dimensions compared to the mean free path and the unique frequency dependence of the dielectric function in metallic silver. The combination of these two factors gives rise to SPR related properties. The size and shape of NPs and the dielectric function of the surrounding medium determines the frequency and resonance strength [36]. The light-interaction cross-sections of AgNPs is dependent on the photon electric field which can extend up to 10 times larger than the geometric cross-sections of the AgNPs. As such, these structures are capable of interaction with light photons that are not physically incident upon them [37,38]. Because of the unique optical characteristics of the AgNPs, non-invasive techniques like dark-field microscopy or surface-enhanced Raman Scattering can be utilized for the inspection of their tracking and cellular uptake [39]. Certain optical characteristics of AgNPs can be modified to obtain interesting results. For example, the absorption spectrum of AgNPs is tunable through careful optimization of the synthesis conditions to the near-infrared region. This eliminates the interference from tissue auto-fluorescence, resulting in NPs which have great promise in tumor-targeting and deep tissue-imaging [40].

### 2.3. Electrical Properties

One of the important electrical properties of AgNPs in a colloidal suspension is zeta potential which is influenced by the size of the NPs. AgNPs possess a large absolute value of zeta potential corresponding to a high surface electrical charge which can result in a strong repulsive force among particles to resist agglomeration [41]. Agglomeration needs to be suppressed at any cost to prevent the growth of the particles from nanoscale to microscale. It has been established that a high zeta potential value of around −25 mV ensures a large enough energy barrier for the stabilization of the nanosuspension. For example, Sankar et al. reported a zeta potential of about −26 ± 0.77 mV for the biosynthesis of AgNPs with an average size of 136 ± 10.09 nm from *Origanum vulgare* [42].

## 3. Biologic Synthesis of AgNPs

Both “top-down” and “bottom-up” approaches can be adopted for NPs synthesis, including AgNPs [43]. A top-down approach involves reducing particle size of an appropriate starting material until NPs are obtained. Different physical and chemical processes like ball milling, chemical etching, laser ablation and sputtering are known top-down approaches [44]. However, a major drawback of top-down approaches is the occurrence of surface defects on the NPs [45]. Conversely, a bottom-up approach produces NPs by assembling smaller entities like atoms, molecules or particles to assemble the NPs [46]. Bottom-up methods are typically chemical or biologic in nature (e.g., sol-gel processes, spray pyrolysis, electrochemical precipitation and laser pyrolysis) [47].

**Figure 1:**
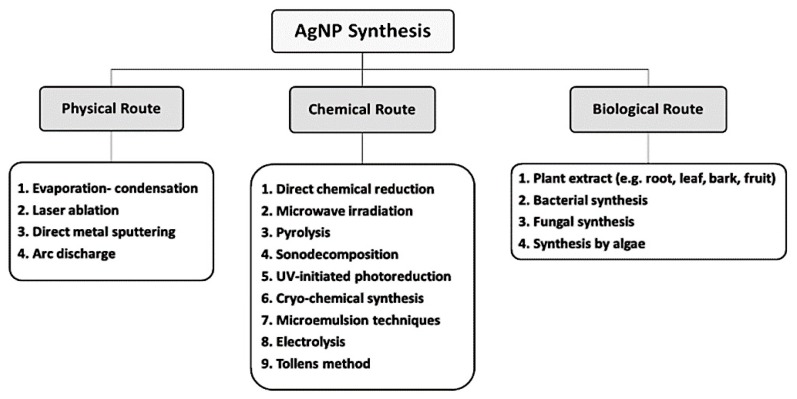
Different routes of AgNPs synthesis.

The downside of most AgNPs synthesis protocols involving chemical and physical methods include associated high cost and the use of toxic and potentially hazardous materials which may pose environmental and biologic threats (Figure 1). NPs produced through chemical synthesis are often not suitable for medical applications because of toxic substances absorbed onto the NPs surfaces. Commercial AgNPs have to be handled and must be cost-effective for practical purposes. Therefore, alternative synthetic methods are required that are environmentally sound and economically feasible. The pursuit of such methods has led to the investigation of biologic processes for synthesis [48]. In biologic synthesis of AgNPs, molecules produced by living organisms (e.g., bacteria, fungi or plants), act as reducing and stabilizing agents [49]. The microbial enzymes or the plant phytochemicals possessing antioxidant or reducing properties are mainly responsible for NP biologic synthesis [50].

### 3.1. Bacterial Synthesis of AgNPs

**Figure 2:**
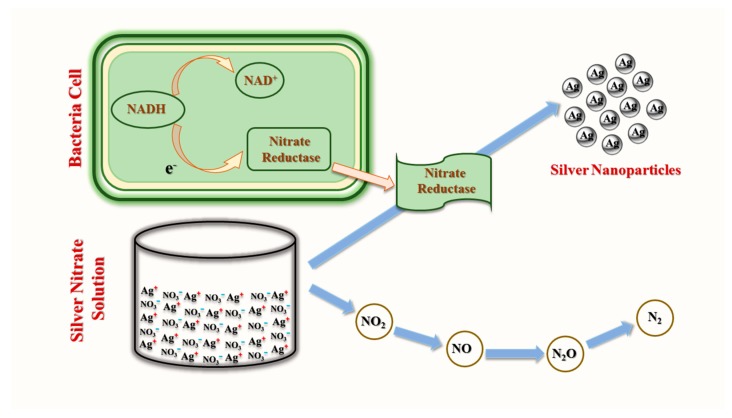
Extracellular enzymatic bacterial synthesis of AgNPs (modified from [54]).

Biosynthesis of NPs may occur extracellularly or intracellularly. A widely utilized extracellular mechanism for AgNPs synthesis via bacteria employs NADH-dependent nitrate reductase-mediated reduction of silver ion (Ag^+^) by several bacteria species, such as *Streptomyces* sp. LK3 [51] and *Bacillus licheniformis* [52]. During reduction, nitrate ions (NO_3_^−^) in silver nitrate (AgNO_3_) are reduced to nitrite (NO_2_^−^) by accepting two protons then releasing two electrons and water. Electrons released in this reaction are transferred to the Ag^+^ ion to form elemental silver (Ag^0^) [53]. This mechanism is possibly dependent on electron transport pathways and enzymatic metal reduction processes (Figure 2).

**Figure 3:**
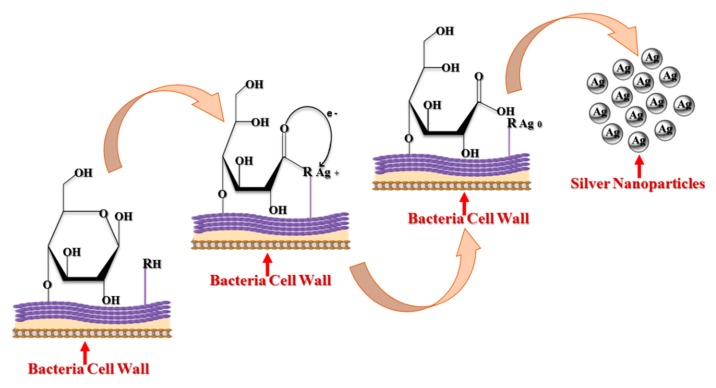
Intracellular non-enzymatic bacterial synthesis of AgNPs (modified from [57]).

However, enzymes are not always necessary for bacterial synthesis of AgNPs. For example, non-enzymatic intracellular synthesis was reported in *Lactobacillus* A09, where Ag^+^ reduction occurred on the bacterial cell surface [53]. Gram-positive bacterial cell walls, such as those of *Lactobacillus* A09, contain numerous anionic surface groups. These groups can provide silver ion biosorption sites [55]. The pH of the surrounding medium decreased slightly after adsorption of Ag^+^ on *Lactobacillus* A09, suggesting competition between proton and silver ion binding to negatively charged sites [56]. Potentially an increase in pH can open bacteria cell wall monosaccharide rings and oxidize these moieties to open-chain aldehydes. At the same time, dissociation of protons from protonated anionic functional groups (–RH) creates negatively charged Ag^+^ adsorption sites on the cell surface. The two electron released while forming the aldehyde group from an alcohol can reduce Ag^+^ ions to elemental Ag^0^ [57]. The steps involved in the bacterial synthesis of AgNPs are mediated through glucose ring-opening (Figure 3).

### 3.2. Fungal Synthesis of AgNPs

**Figure 4:**
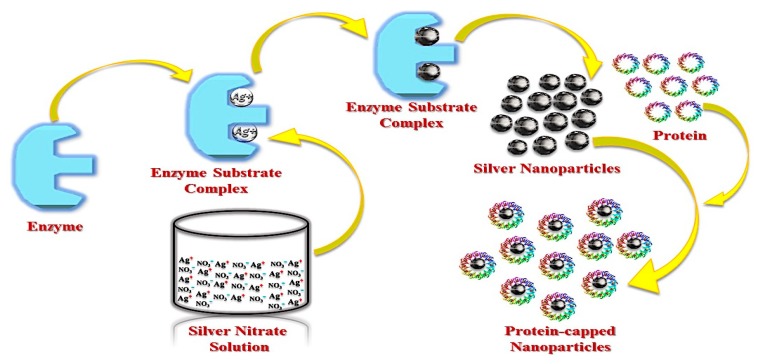
Fungal synthesis of AgNPs (modified from [60]).

Due to their inherent ability to efficiently secrete protein, fungi can produce substantially larger amounts of AgNPs than bacteria [58]. However, the exact mechanism by which fungi synthesize AgNPs is not completely understood. Mukherjee et al. hypothesized that the following steps may occur during synthesis in *Verticillium* model system: Ag^+^ ions interact electrostatically with negatively charged carboxylate groups in the mycelial cell wall and are trapped at the cell surface. Subsequently, trapped ions are reduced by specific fungal enzymes [46]. Enzymes involved in fungal NP synthesis are generally reductases [59]. For example, during synthesis of AgNPs by *Aspergillus flavus*, a 32-kDa reductase protein secreted by the fungal isolate, reduces Ag^+^ ions [60]. A schematic representation of AgNPs synthesis by fungi is presented in Figure 4.

### 3.3. Plant Synthesis of AgNPs

Plant extracts have been used for AgNPs synthesis and can be superior to bacterial and fungal synthetic systems due to their availability, low toxicity and safety. A wide range of extracted phytochemicals can rapidly reduce silver ions. Reduction can be rapid compared to microbial fermentation [50], as this approach can avoid lengthy sterile cell-culture under controlled conditions [61]. Furthermore, plant extracts can act as both reducing and stabilizing agent during AgNPs synthesis. Approaches of reduction with plant extracts can be applied to other metallic NPs [62]. The plant source determines the properties of the AgNPs [63]. The concentration of individual phytochemicals and combinations of phytochemicals present vary considerably in extracts depending on the plant source. Therefore, AgNPs’ properties can be selected by controlling the extract composition [64]. The biosynthesis of AgNPs by plant extracts commonly involves mixing of an aqueous solution of silver nitrate with an aqueous plant extract. Typical reactions take place at room temperature and requires only a few minutes to complete [47].

**Figure 5:**
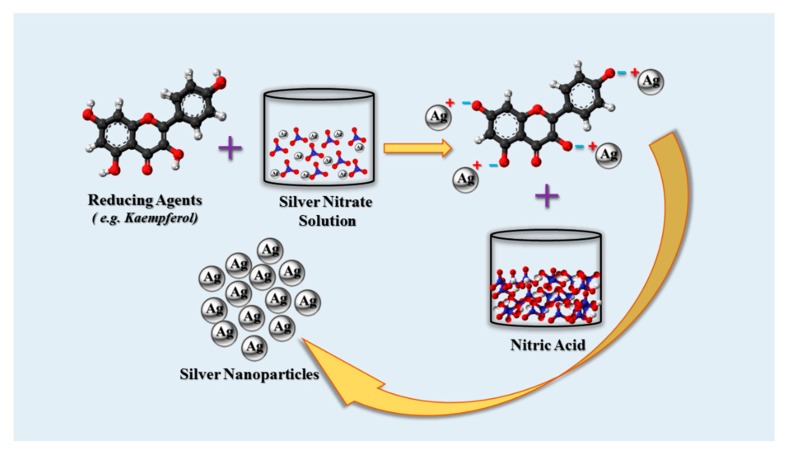
Synthesis of AgNPs by reaction of AgNO_3_ with phytochemicals (concept of this representation was based on [67]).

Numerous phytochemicals have been identified that are capable of producing AgNPs. As examples, flavonoids, terpenoids, terpenes, flavones, phenolics, polysaccharides, saponins, tannins and alkaloids have all been used for AgNPs synthesis [47,65]. Phytochemical functional groups, such as hydroxyl, aldehyde, ketone, carboxyl and amino groups are capable of reducing Ag^+^ ions [50,66]. Due to the diversity of phytochemicals the exact mechanism of AgNPs synthesis varies. However, the chief mechanism is the reduction of Ag^+^ ions by specific functional groups. For example, *Punica granatum* peel extract contains the flavonoids- kaempferol and naringin along with their glycosides. All of these compounds have hydroxyl (–OH) groups that cause the reduction of the Ag^+^ ions, resulting in the formation of AgNPs [67]. A typical reduction is possible by the use of kaempferol as a reducing agent (Figure 5).

Interestingly, not all phytochemicals present in plant extracts capable of reducing other compounds remain active during Ag^+^ ions reduction and only certain phytochemicals are involved in this reduction. For example, phytochemical screening revealed the presence of phenols, glycosides, flavonoids, reducing sugars, resins, tannins, terpenoids, steroids, alkaloids and saponins in *Anagallis tenella* leaf extract (Table 1).

However, it was discovered that only flavonoids were involved in AgNPs synthesis [65]. Active phytochemicals associated with AgNPs synthesis in other plants are listed in Table 1. As stated earlier, the wide variety of phytochemicals present in different plants that contribute to AgNPs synthesis complicates this field of study. Each plant has a unique spectrum of phytochemicals that act as organic reducing agents [72].

## 4. AgNPs Characterization

Ultraviolet-visible (UV-Vis) spectroscopy, dynamic light scattering (DLS), scanning electron microscopy (SEM), transmission electron microscopy (TEM), Fourier-transform infrared spectroscopy (FTIR), thermogravimetric analysis (TGA), X-ray diffraction (XRD) and energy dispersive spectroscopy (EDS) are commonly used for AgNP characterization [75,76].

UV-Vis light spectroscopy is used to determine AgNPs formation by measuring the optical absorbance spectra. The wavelength of the light absorbed is heavily affected by the size and aspect ratio of these NPs. Variations in sizes cause each solution to have a different color. The vibrant colors occur because the conduction electrons on the surface of each nanoparticle vibrate when a particular wavelength of light excites them (“surface plasmon resonance” phenomenon as discussed earlier). These vibrations result in extremely bright colors which can be tweaked by altering the particle size and shape. In case of spherical NPs, the larger they are, the “bluer” they appear. The color change results from the interaction of the individual metal atoms with each other [77].

DLS is a technique that can be utilized to determine particle size and particle size distribution [78]. Since the range of size DLS is capable of measuring is a few nanometers to a few micrometers, it is a suitable method for measuring the size of AgNPs. This method operates by calculating the change in the light frequency while interacting with the particles of varying size. The smaller the particles are, the greater is the shift in the light frequency [79].

SEM and TEM can be utilized to characterize AgNPs shape and morphology [80]. It has been demonstrated that mostly spherically shaped NPs are detected by electron microscopy in case of plant-mediated AgNPs [81]. TEM has a thousand-fold higher resolution than SEM but SEM images have superior depth of field [82]. These two modes of electron microscopy are complementary to each other.

FTIR spectroscopy has been used to characterize AgNPs surface chemistry, i.e., the functional groups present on NPs surfaces can be identified with the help of this technique [83]. The surface-bound functional groups illustrate the capping and reducing agents involved in AgNPs biosynthesis. Identifying the type of capping agent is vital because the type of capping agent regulates the efficacy of the AgNPs [84]. The capping agents can stabilize the AgNPs by preventing the agglomeration of the particles. In addition, they inhibit the interactions of the particles with in vivo components and restores their activities [40]. In the FTIR analysis spectra, major peaks are identified; each peak corresponds to a specific functional group. For example, the prominent peak at 3348 cm^−1^ is attributed to O–H stretching, whereas the peaks at 2974 and 2887 cm^−1^ correspond to C–H stretching vibrations of methyl, methylene or methoxy groups. The other noticeable peaks positioned at 1656 and 420 cm^−1^ are attributed to the C=O stretching in carbonyl group, while the peak at 1049 cm^−1^ is attributed to the C=O stretching of the alcoholic group [85].

The types of bio-organic compounds or functional groups on the AgNPs surfaces can be further confirmed by TGA analysis. TGA identifies the organic compounds on the NPs surfaces by determining the thermal stability of the compounds. TGA detects the characteristic weight losses at certain ranges of temperatures which is attributes to specific compounds on the AgNPs surfaces. For example, Kajani et al. reported that the weight loss of AgNPs synthesized using ethanolic extract of *T. baccata* was 27% when the temperature of the sample was raised from 0–521 °C. Another weight loss of 8.3% followed when the temperature reached 678 °C. The final result was a total weight loss of about 36.89% after increasing the temperature up to 700 °C [40].

Phase identification and crystal structure determination of NPs can be determined by X-ray diffraction of monochromatic collimated X-rays (XRD) by NPs [86]. With the help of XRD, the presence of AgNPs in the synthesis product can be confirmed. The confirmation takes place by identifying the peaks in the XRD spectrum characteristic of the face centered cubic crystal structure of metallic silver. The diffraction peak values at 38.39°, 44.53°, 64.13° and 77.59° are referred to (111), (200), (220) and (311) lattice planes, respectively of metallic silver. Moreover, the average grain size of the AgNPs can be computed from the X-ray diffraction (XRD) analysis by using Scherer equation shown below:

$$D=\frac{K\lambda }{\beta cos\theta }$$

where ‘*D*’ is the particle diameter size, ‘*K*’ is a constant, ‘*λ*’ is the wavelength of X-ray source, ‘*β*’ is the full width half maximum (FWHM) and ‘θ’ is the diffraction angle [85].

Elemental analysis of NPs can be performed by EDS [87]. In the case of AgNPs, the energy dispersive spectroscopy (EDS) profile displays a strong signal corresponding to silver fluorescence. However, fluorescence from other elements, such as carbon and oxygen—originating from biomolecules attached to the AgNPs—can also appear [81].

## 5. Biosynthesized AgNPs as Anticancer Agents in Vitro

Optimal procedures for anti-cancer AgNPs preparation are broadly being examined. It is worth mentioning that we have noted some biosynthesized AgNPs from bacterial and fungal sources in the Table S1 to keep the review less verbose. However, AgNPs synthesized with the contribution of biologic sources displayed significant inhibitory activities against the viability of certain cancerous cell lines. In those processes, for example, the bio-extracts collected from different plant parts (e.g., leaf, root, flower or fruit) were used as reducing agents.

Literature searches have uncovered studies of 23 plant extracts which were used for AgNPs preparation. Many of these AgNPs were either toxic to the breast cancer cell line MCF-7 or inhibited its growth. The sizes of NPs reported in these studies ranged from 5–80 nm; their shapes also varied (e.g., spherical, cuboidal, pentagonal and hexagonal). The IC_50_ values of these extracts against Michigan Cancer Foundation (MCF)-7 cells ranged from 3.04–250 μg/mL (Table 2). Moreover, some research outcomes showed that the half maximal inhibitory concentration (IC_50_) of AgNPs was dependent upon the dose of the extract given [40,65,75,85,88,89,90,91,92,93,94,95,96,97,98,99,100,101,102,103,104]. Data regarding the breast cancer cell line MCF-7 cells are enumerated in Table 3 with citations.

Biosynthesized AgNPs have also been found to inhibit the brain cancer cell line HNGC2 [88]. These spherical AgNPs ranged in size from 20–80 nm, and the IC_50_ values were dose-dependent [101]. Biosynthesized AgNPs have also shown notable inhibitory activity against the cervical cancer cell lines Siha and HeLa. Growth of the Siha cancer cell line was inhibited by 2–18 nm triangular and hexagonal AgNPs, with an IC_50_ of ≤4.25 μg/mL [106]. In contrast, spherically shaped AgNPs, with sizes ranging between 5–120 nm, achieved inhibitory effects on HeLa cancer cell lines. The IC_50_ values varied depending on the preparation method of the AgNPs and also depended upon the plant extracts used [23,25,95,107,108,109,110,111,112]. The bio-synthesized AgNPs, inhibited four colon cancer cell lines (e.g., COLO 205, HCT 15, HCT-116 and HT29 cells), with IC_50_ values ranging from 5.5–100 μg/mL [61,103,105,113,114,115,116]. The particles were substantially spherical in shape, with sizes ranging from 7.39–80 nm. Available data regarding these AgNPs in the context of brain, cervical and colon cancers are represented in Table 3.

Cuboidal and spherical bio-AgNPs with sizes ranging from 59–94 nm, prepared from different plant extracts, inhibited the A431 cell line, an epidermoid carcinoma, with IC_50_ values ranging from 78.58–83.57 μg/mL [117]. Inhibition by bio-extract-derived spherical AgNPs with sizes ranging from 5–50 nm was also observed against the AGS cell line, a gastric carcinoma, with an IC_50_ of 21.05 μg/mL [118]. A hepatic cancer cell line (Hep-G2) was inhibited by spherical AgNPs that ranged in size from 6.4–1200 nm. In those studies, the results were presented according to different parameters; IC_50_ values ranged from 10.02 to more than 30 μg/mL, the load of detection was 31.25 ng/mL, there was 13.86% viability at 25 μL, and some of the other results demonstrated a dose dependent relationship [75,89,97,119,120]. Spherical bio-AgNPs that were less than 20 nm in size inhibited the Caco-2 intestinal cancer cell line, where the IC_50_ value was greater than 30 μg/mL. Biosynthesized, spherical AgNPs with sizes less than 40 nm blocked the Hek-293 kidney cancer cell line in a dose-dependent manner [95,98]. The 31-nm, spherical, green AgNPs repressed the Hep-2 laryngeal carcinoma cell line, with IC_50_ values of 3.42 μg/mL and 12.5 μg/mL, and an AgNPs concentration of 500 nM [105,121,122]. The H1299 and HL-60 leukemia cell lines were inhibited by 8–22 nm spherical bio-AgNPs with an IC_50_ value of 5.33 μg/mL and in a dose-dependent manner, respectively [112,123]. The data related to epidermoid carcinoma, laryngeal carcinoma, gastronomic carcinoma, hepatic cancer, intestinal cancer and kidney cancer are enumerated in Table 4.

According to a literature review, 13 bio-extracts have been successfully used as reducing agents to prepare AgNPs that can block the activities of the A549 lung cancer cell line [42,75,89,101,104,125,126,127,128,129,130,131]. The size of those spherically shaped NPs ranged from 13–136 nm, and the inhibitory actions were observed to follow a dose-dependent relationship, with various IC_50_ and LD_50_ values as mentioned below [42,75,89,101,104,125,126,127,128,129,130,131]. The spherical bio-AgNPs with sizes ranging from 5–50 nm showed inhibitory activities against the lymphoma Jurkat cell line, and the IC_50_ was 21.05 μg/mL [132]. The B16F10 melanoma cell line was inhibited by the 20–228 nm spherical bio-AgNPs, with an IC_50_ value of 7.6 ± 0.8 μg/mL, following a dose-dependent relationship [101,133]. The 100–120 nm flower-shaped AgNPs exhibited notable repressive actions against the KB oral cancer cell line, where the IC_50_ was 0.6 μg/mL [134]. The 15–32 nm spherical bio-AgNPs impeded the PA1 ovarian cancer cell line, with an IC_50_ value of 7.5 μg/mL and in a dose-dependent manner [125,127]. Inhibitory activities were observed against the BxPC 3 pancreatic cancer cell line by spherical bio-AgNPs with sizes ranging from 8–22 nm, and the IC_50_ was 38.9 μg/mL [123]. The 9–99 nm and 8–22 nm spherical AgNPs repressed the PC3 and VCaP prostate cancer cell lines, respectively, in dose-dependent manners with IC_50_ values ranging from 6.85–87.33 μg/mL [76,123,135]. All of these data regarding the cell lines mentioned above for lung cancer, lymphoma, melanoma, oral cancer, ovarian cancer, pancreatic cancer and prostate cancer are summarized in Table 5 with appropriate citations.

## 6. Anticancer Mechanism of Biosynthesized AgNPs

The cytotoxicity of AgNPs depends upon their size and shape. For example, it was reported that AgNPs with diameters of 100–160 nm, lengths of 1.5–25 μm, and spherical shapes (30 nm) showed potential cytotoxic effects on human lung epithelial A549 cells [42]. The most probable reason for this is that within this size and shape range, the AgNPs can directly contact the cell surfaces and initiate cytotoxicity [137]. NPs are safe at lower dose and can be toxic at higher dose. Usually cells treated with various NPs concentration show a dose-dependent increase in cell inhibition. AgNPs in different formulations exhibit variable dose effects that may affect the cytotoxicity or improve the anticancer activity. Many reports demonstrated the anticancer activity of AgNPs synthesized by biosynthetic routes. Nevertheless, AgNPs synthesized by biosynthesis also show sort of cytotoxicity [138,139]. The sizes and shapes of the different AgNPs reviewed in this manuscript are summarized in Table 2, Table 3, Table 4 and Table 5. Furthermore, most of the NPs that were synthesized from plant sources were spherical in shape and demonstrated significant efficacy against cancer cell lines (Table 2, Table 3, Table 4 and Table 5).

**Figure 6:**
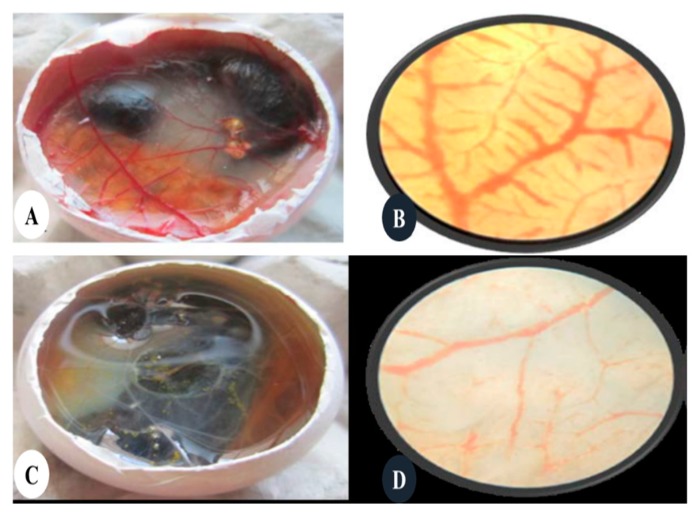
AgNPs were evaluated for their effects on the process of angiogenesis in a chicken embryonic chorioallantoic membrane model. (**A**) control egg, (**B**) microscopic view of A, (**C**) egg treated with AgNPs, (**D**) microscopic view of (**C**) (Adapted from Khandia et al. with permission [144]).

Blood vessels play a vital role in supplying nutrients and clearing waste products at the tissue level; the formation of new blood vessels from existing vessels is termed angiogenesis. Wound healing and granulation tissue formation are some potential results of angiogenesis [140]. According to Folkman’s hypothesis, the growth of solid tumors is the result of new blood vessel formation [141]. This process is thought to play a pivotal role in the growth and spread of cancer and is regulated by both activator and inhibitor molecules. According to this hypothesis, blood supply is necessary for the growth of tumors. The new blood vessels contribute to tumor growth by supplying oxygen and nutrients to cancer cells helping them to invade and spread throughout the body; this phenomenon is known as metastasis [142]. Recent studies have suggested that green-synthesized AgNPs displayed efficacy in treating retinal neovascularization (RNV)-like diseases. They inhibited vascular endothelial growth factor-induced RNV and blocked extracellular signal-related kinase (ERK½) activation via regulation of vascular endothelial growth factor receptor-2 phosphorylation. These anti-angiogenesis properties have been applied in cancer treatment using different approaches of AgNPs [143]. Interestingly, Rekha Khandia and her team successfully reduced the angiogenesis in embryonated chicken mode (Figure 6).

**Figure 7:**
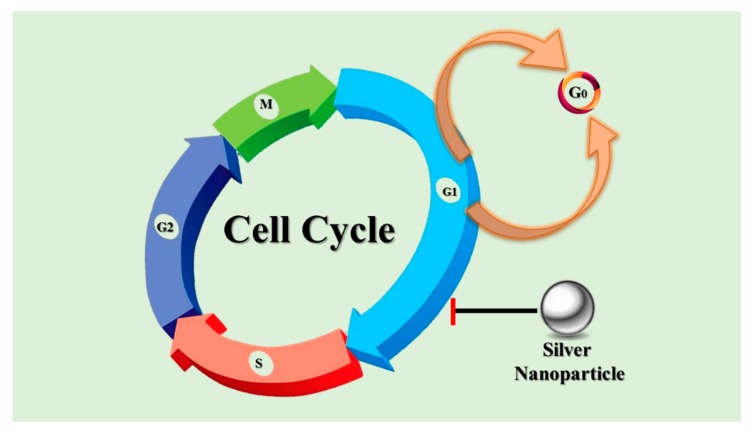
Schematic diagram of cell cycle arrest by AgNPs.

The cell cycle incorporates a complex series of signaling pathways, by which a cell grows replicates its DNA and divides. This phenomenon plays a vital role in cancer progression. However, due to the genetic mutations that occur in cancer, this regulatory process malfunctions, which leads to uncontrolled cell proliferation. DNA synthesis (S), Gap2/mitosis (G2/M), Gap1 (G0/G1) and subG1 are all vital checkpoints for cell cycle arrest [145] in Figure 7. Different studies have suggested that green-synthesized AgNPs can arrest the sub-G1 phase of the cell cycle. Chang et al. revealed that the sub-G1 phase was arrested in curcumin-treated cancer cells, which explains the direct correlation between the sub-G1 phase arrest of cancerous cells and apoptosis [146]. Another study demonstrated the connection between an increased cancer cell population in the sub-G1 phase and expression of the pro-apoptotic protease caspase-3 [147].

## 7. Toxicity

The toxicity of AgNPs has been mentioned in different studies; Bharadwaj Punita reported that AgNPs could be highly toxic to mammalian cells, e.g., brain cells, liver cells and stem cells [148]. Besides, Mahmoudi and his team stated that the AgNPs are highly toxic to healthy/normal cells [149]. Conversion of metal into its nano-form may bring the risk of toxicity. However, the green synthesis method reduces the toxicity of AgNPs. The nominal toxicity mainly depends upon its coating. The coating of AgNPs stabilize the particles and prevent their agglomeration. Here, the biocompatible behavior of coating makes green synthesized AgNPs suitable for numerous medicinal applications [150].

## 8. Inter-Connection between Antimicrobial Property and Anticancer Activity of AgNPs

Studies have revealed that the mitochondrial activation and reactive oxygen species (ROS) overproduction are the key factors of AgNPs in antimicrobial and anticancer defense. Interestingly, the ROS production and the subsequent damages resulting from oxidative stress are AgNPs size-dependent, smaller NPs cause better ROS overproduction [151]. AgNPs can induce mitochondrial chain and complex disruption which lead to superoxide anion leakage [152,153]. Here, Ag+ ions are released that can influence the mitochondrial enzymes and also interact with –SH groups of proteins and glutathione (GSH). Because of this situation, the ROS scavenging potential of GSH decreases and oxidative stress takes place [154]. DNA damage could change the gene expression, and cellular death may be exhibited as apoptosis [153,154].

## 9. Clinical Application

AgNPs have been investigated intensively regarding their promising anticancer effects exhibited in different human cancer cell lines, such as endothelial cells, IMR-90 lung fibroblasts, U251 glioblastoma cells and MDA-MB-231 breast cancer cells [40,106,155,156]. AgNPs showed great promises as effective anti-tumor drug-delivery systems [157]. As previously mentioned, conventional cancer treatments such as chemotherapy, radiotherapy or surgery have their limitations associated with drug toxicity, unpredictable side effects, drug resistance problems and lack of specificity. AgNPs overcome these disadvantages by reducing the side effects and enhancing the efficiency of cancer therapy. One of their distinguishing features is the ability to cross various biologic barriers and to provide targeted delivery of drugs. Green synthesis of AgNPs together with specific delivery of anticancer drugs to tumor tissues offers an innovative approach for improving cancer treatment. Currently, theranostics approach (the combination of therapy and diagnosis) is one of the most attractive and challenging approaches, which is effective in personalized therapy for cancer treatment. As mentioned in Section 2.2, AgNPs are plasmonic structures, particularly capable of scattering and absorbing the lights impinging certain areas. After their selective uptake into cancerous cells, AgNPs-derived scattered lights can be used for imaging purposes. Silver has been used for centuries in dental care as a major component of amalgams used for tooth restoration. However, AgNPs proved to be efficient agents in dental practice; nevertheless, they remain controversial candidates due to their variable toxicity in biologic systems [158]. Interestingly, AgNPs showed promising activity against both the malarial parasite (*Plasmodium falciparum*) and its related vector (*Anopheles* female mosquito) [159].

Bioavailability of AgNPs is low in oral, dermal and inhalational exposures, however, it depends on the particle size, dose, surface coating and soluble fraction. For examples, Park and his team revealed that in a single oral exposure in rats, the bioavailability of citrate-coated 7.9 nm AgNPs was 1.2% and 4.2% in the 1 and 10 mg/kg, respectively [160]. After oral exposure, AgNPs can be ionized to form Ag+ in the stomach, but the dissolution is incomplete due to the limited gastric residence time (10–240 min). Ag^+^ and other soluble complexes like AgCl(aq) and AgCl_2_^−^ can be absorbed by the gastrointestinal tract into the systemic circulation, where Ag+ can bind to proteins with thiol groups such as serum albumin and small thiol molecules like glutathione (GSH). These complexes generate H+ and GS-Ag which then produce Ag-GSH complexes that are distributed throughout the body. However, biliary pathway is the major elimination route for AgNPs [161].

## 10. Future of AgNPs

Despite all the recent advancements in cancer treatment, cancer remains one of the most common causes of death around the globe. It is already known to us that the conventional treatment strategies often have many side effects of their own. Therefore, scientists are looking to design novel strategies for the diagnosis and treatment of cancer. Recently, green synthesis of AgNPs has gained much attention in the pharmaceutical field. The use of green chemistry is non-toxic, cheap and environmentally friendly, although there are some disadvantages of biologic methods as listed in Box 1. The high biodegradability and clearance of AgNPs also play a pivotal role in avoiding the potential effect of long-term toxicity. The AgNPs showed great promises in cases of nanomedicine-based treatment. However, clinical trials of AgNPs-based nanomedicine are necessary for guiding the future direction of their application. Currently, investigations into the biodegradability, dose and route of administration are the major hurdles that need to be tackled in clinical trials. Moreover, AgNPs can be used as a vital cancer cell visualization and detection tool in diagnosing cancer at its early stages [162]. It has already been shown that green synthesis of AgNPs can help with in vivo fluorescent tumor imaging [123]. We believe that green-synthesized AgNPs will be used as potential cancer therapeutics and diagnostics agents in the upcoming era of cancer treatment.

Box 1 **Advantages and disadvantages of green synthesis methods.**

Advantages of biologic methods:

1. These methods are inexpensive, eco-friendly and non-toxic.
2. No complex setup is required to conduct the synthesis process.
3. It is not necessary to use stabilizing agents to prevent agglomeration of the NPs.
4. Since these processes are carried out in ambient conditions, they are not energy intensive.
5. These methods offer finer tuned control of the size and shape of the NPs compared to chemical and physical methods.

Disadvantages of biologic methods:

1. Synthesis by biologic methods is not as fast as synthesis by chemical methods.
2. Because of the presence of numerous biomolecules present in the biologic sources, it is difficult to pinpoint the exact biomolecules responsible for the synthesis of the NPs.
3. If the biologic synthesis is conducted on a large scale, an ecological imbalance may kick in from overuse of different biologic species.
4. Microbe toxins may be brought together by biologic synthesis.

## 11. Conclusions

Business experts have extrapolated that the global market of nanotechnology has a potentially bright future. Commercially produced AgNPs are being manufactured at a significantly large scale and the application of NPs as therapeutic agents is increasing. AgNPs are promising drug leads based on the successful results of previous research studies as well as their cost. However, there are some basic hindrances to using the AgNPs as therapeutic agents in terms of toxicity. To overcome these hindrances—and for use in preclinical trials on humans or any other living bodies—the AgNPs should be biocompatible, non-toxic and free from side effects. Finally, usage of biogenic AgNPs as cancer nanomedicine will require elaborate datasets that uncover potential toxicity and pharmacological issues such as the side effects.

## Figures and Tables

**Table 1:** Active phytochemical fractions associated with AgNPs synthesis in specific plants.

Plant	Phytochemical	Reference
Alternanthera tenella	Flavonoids	[65]
Cocos nucifera	Carbohydrates, alkaloids, terpenoids, tannins, saponins, phenolics and reducing sugars	[68]
Lemongrass	Reducing sugars	[69]
Ocimum sanctum	Caffeine	[70]
Chrysanthemum indicum	Tannins, flavonoids and glycosides	[71]
Dalbergia spinose	Reducing sugars and flavonoids	[72]
Cinnamomum camphora	Phenolics, terpenoids, polysaccharides and flavones	[73]
Eucalyptus hybrid	Flavonoids and terpenoids	[74]

**Table 2:** Studies AgNPs from plants that inhibit the breast cancer cell line MCF-7.

Plant	Part Used	NP Size/NP Shape	Incubation Time	IC_50_	Ref.
*Achillea biebersteinii*	Flower	12 nm/spherical, pentagonal	3 h	20 μg/mL	[88]
*Alternanthera sessilis*	Aerial parts	10–30 nm/spherical	6 h	3.04 μg/mL	[94]
*Alternanthera tenella*	Leaf	About 48 nm/-	-	42.5 μg/mL	[65]
*Andrographis echioides*	Leaf	68.06 nm/cubic, pentagonal, hexagonal	12 h	31.5 μg/mL	[91]
*Azadirachta indica*	Leaf	<40 nm/spherical	6 h	Dose dependent	[95]
*Butea monosperma*	Leaf	20–80 nm/spherical	2 h	Dose dependent	[101]
*Coriandrum sativum*	Leaf	About 37 nm/spherical, rod, triangular, hexagonal	-	30.5 μg/mL	[97]
*Citrullus colocynthis*	Leaf	13.37 nm/spherical	24 h	>30 μg/mL	[103]
	Roots	7.39 nm/spherical	24 h	2.4 μg/mL	
	Seeds	16.57 nm/spherical	24 h	>30 μg/mL	
	Fruit	19.26 nm/spherical	24 h	>30 μg/mL	
*Dendrophthoe falcata (L.f) Ettingsh*	Mistletoe, leaf	5–45 nm/spherical	24 h	Dose dependent	[100]
*Erythrina indica*	Root	20–118 nm/spherical	Overnight	23.89 (% viability at 25 μL)	[75]
*Melia dubia*	Leaf	7.3 nm/irregular	15 min	31.2 μg/mL	[85]
*Olax scandens*	Leaf	30–60 nm/spherical	2 h	Dose dependent	[96]
*Panax ginseng*	Leaf	-	-	Dose dependent	[89]
*Piper longum*	Fruit	46 nm/spherical	24 h	67 μg/mL	[99]
*Quercus (genus)*	Fruit hull	40 nm/spherical	-	50 μg/mL	[92]
*Rheum emodi*	Root	27.5 nm/spherical	24 h	Dose dependent	[102]
*Sesbania grandiflora*	Leaf	22 nm/spherical	24 h	20 μg/mL	[93]
*Solanum trilobatum*	Fruit	41.90 nm/spherical, polygonal	-	30 μg/mL	[98]
*Syzygium cumini*	Flower	<40 nm/spherical	6 h	Dose dependent	[95]
*Syzygium aromaticum*	Fruit	5–20 nm/spherical	20 min	70 µg/mL	[104]
*Tabernae montana divaricate*	Leaf	Mean 22.85 nm/spherical	24 h	20 μg/mL	[90]
*Taxus baccata*	Needles	Mean 75.1 nm/spherical	48 h	0.25 mg/mL	[40]
*Ulva lactuca*	Whole	56 nm/spherical	10 min	37 µg/mL	[105]

**Table 3:** Studies of plant extracts for the biosynthesis of anticancer AgNPs (brain, cervical and colon cancers cell lines).

Cancer	Plant	Part Used	NP Size/NP Shape	Incubation Time	Cell Line	IC_50_	Ref.
**Brain Cancer**	*Butea monosperma*	Leaf	20–80 nm/spherical	2 h	HNGC2	Dose dependent	[101]
**Cervical Cancer**	*Azadirachta indica*	Leaf	2–18 nm/triangular, hexagonal	-	Siha	≤4.25 μg/mL	[106]
	*Acorous calamus*	Rhizome	31.86 nm/spherical	20 h	HeLa	Dose dependent	[107]
	*Azadirachta indica*	Leaf	<40 nm/spherical	6 h		Dose dependent	[95]
	*Calotropis gigantea*	Latex	5–30 nm/spherical	24 h		Dose dependent	[108]
	*Cymodocea serrulata*	Whole	17–29 nm/spherical	2 h		107.7 (GI_50_)	[109]
	*Heliotropium indicum*	Leaf	80–120 nm/spherical	2 h		20 μg/mL	[110]
	*Melia azedarach*	Leaf	78 nm/cubical, spherical	10 min		300 μg/mL (LD_50_)	[23]
	*Moringa olifera*	Stem bark	38–40 nm/spherical, pentagonal	24 h		Dose dependent	[25]
	*Podophyllum hexandrum*	Leaf	14 nm/spherical	30–150 min		20 μg/mL	[111]
	*Sargassum vulgare (algae)*	Whole	10 nm/spherical	3 h		Dose dependent	[112]
	*Syzygium cumini*	Flower	<40 nm/spherical	6 h		Dose dependent	[95]
**Colon Cancer**	*Plumeria alba*	Flower	36.19 nm/spherical	30 min	COLO 205	5.5 μg/mL	[113]
	*Rosa indica*	Petal	23.52–60.83 nm/spherical	1 h	HCT 15	30 μg/mL	[114]
	*Vitex nigundo*	Leaf	22 nm/spherical	4 h		20 μg/mL	[115]
	*Citrullus colocynthis*	Leaf	13.37 nm/spherical	24 h	HCT-116	>30 μg/mL	[103]
		Roots	7.39 nm/spherical	24 h		>30 μg/mL	
		Seeds	16.57 nm/Spherical	24 h		>30 μg/mL	
		Fruit	19.26 nm/spherical	24 h		21.2 μg/mL	
	*Commelina nudiflora L.*	Whole	24–80 nm/spherical, triangular	24 h		100 μg/mL	[61]
	*Gymnema sylvestre*	Leaf	-/spherical	24 h	HT29	85 μg/mL	[116]
	*Ulva lactuca (algae)*	Whole	56 nm/spherical	10 min		49 μg/mL	[105]

NP—nanoparticle.

**Table 4:** Studies using plant extracts for the biosynthesis of AgNPs that inhibited epidermoid, laryngeal, gastronomic, hepatic, intestinal and kidney cancers cell lines.

Cancer	Plant	Part Used	NP Size/NP Shape	Incubation Time	Cell Line	IC_50_	Ref.
Epidermoid Cancer	*Acorus calamus*	Rhizome	59 nm/cuboidal, spherical	5–60 min	A431	78.58 μg/mL	[117]
	*Cucurbita maxima*	Petal	76 nm/cuboidal, spherical	5–60 min		82.39 μg/mL	
	*Moringa oleifera*	Leaf	94 nm/cuboidal, spherical	5–60 min		83.57 μg/mL	
Gastric Cancer	*Artemisia marschalliana*	Aerial Part	5–50 nm/spherical	5 min	AGS	21.05 μg/mL	[118]
Hepatic Cancer	*Allium sativum*	Whole	100–1200 nm/spherical	48 h	HEP-G2	31.25 ng/mL (LD_50_)	[119]
	*Citrullus colocynthis*	Leaf	13.37 nm/spherical	24 h	Hep-G2	10.02 μg/mL	[97]
		Root	7.39 nm/spherical	24 h		17.2 μg/mL	
		Seed	16.57 nm/spherical	24 h		>30 μg/mL	
		Fruit	19.26 nm/spherical	24 h		22.4 μg/mL	
	*Erythrina indica*	Root	20–118 nm/spherical	Overnight		13.86 (% viability at 25 μL)	[75]
	*Panax ginseng*	Leaf	-	-		Dose dependent	[89]
	*Rubus glaucus Benth*	Leaf	12–50 nm/Quasi-spherical	48 h		Dose dependent	[120]
Intestinal Cancaer	*Taxus yunnanensis*	Callus	6.4–27.2 nm/spherical	10 min	SMMC-7721	27.75 μg/mL	[124]
Intestinal Cancaer	*Citrullus colocynthis*	Leaf	13.37 nm/spherical	24 h	Caco-2	>30 μg/mL	[97]
		Root	7.39 nm/spherical				
		Seed	16.57 nm/spherical				
		Fruit	19.26 nm/spherical				
Kidney Cancer	*Azadirachta indica*	Leaf	<40 nm/spherical	6 h	Hek-293	Dose dependent	[95]
	*Syzygium cumini*	Flower	<40 nm/spherical	6 h		Dose dependent	[98]
Laryngeal Cancer	*Citrullus colocynthis*	Callus	31 nm/spherical	24 h	Hep-2	3.42 μg/mL	[121]
	*Suaeda monoica*	Leaf	31 nm/spherical	5 h		500 nM, AgNPs conc.	[122]
	*Ulva lactuca (algae)*	Whole	56 nm/spherical	10 min		12.5 μg/mL	[105]
Leukemia Cancer	*Dimocarpus longan*	Peel	8–22 nm/spherical	2 h	H1299	5.33 μg/mL	[123]
	*Sargassum vulgare (algae)*	Whole	10 nm/spherical	3 h	HL-60	Dose dependent	[112]

NP: nanoparticle.

**Table 5:** List of studies using plant extracts for the biosynthesis of AgNPs that inhibited cell lines of lung cancer, lymphoma, melanoma, oral cancer, ovarian cancer, pancreatic cancer and prostate cancers.

Cancer	Plant	Part Used	NP Size/NP Shape	Incubation Time	Cell Line	IC_50_	Ref.
Lung Cancer	*Acorous calamus*	Rhizome	31.86 nm/spherical	20 h	A549	Dose dependent	[106]
	*Artemisia princeps*	Leaf	20 nm/spherical	15 min		Time dependent	[125]
	*Butea Monosperma*	Leaf	20–80 nm/spherical	2 h		Dose dependent	[101]
	*Croton bonplandianum*	Leaf	32 nm/spherical	1 h		Dose dependent	[128]
	*Cymodocea serrulata*	Leaf	29.28 nm/spherical	1 h		100 μg/mL (LD_50_)	[130]
	*Gossypium hirsutum*	Leaf	13–40 nm/spherical	3 min		40 μg/mL	[126]
	*Olax scandens*	Leaf	30–60 nm/spherical	2 h		Dose dependent	[75]
	*Origanum vulgare*	Leaf	136 ± 10.09 nm/spherical	Temp. dependent		100 μg/mL (LD_50_)	[42]
	*Panax ginseng*	Leaf	-	-		Dose dependent	[89]
	*Penicillium decumbens (MTCC 2494)*	Whole	30–60 nm/spherical	-		80 μg/mL, 24 h, 60 μg/mL, 48 h	[129]
	*Rosa damascena*	Petal	15–27 nm/spherical	0–25 min		80 μg/mL	[131]
	*Scoparia dulcis*	Leaf	15–25 nm/spherical	1 h		Dose dependent	[127]
	*Syzygium aromaticum*	Fruit	5–20 nm/spherical	20 min		70 µg/mL	[104]
Lymphoma Cancer	*Abelmoschus esculentus*	Pulp	~6.7 nm/spherical	27 h	Jurkat	16.15 μg/mL	[132]
Melanoma Cancer	*Excoecaria agallocha L.*	Leaf	228 nm/-	-	B16F10	7.6 ± 0.8 μg/mL	[133]
	*Butea monosperma*	Leaf	20–80 nm/spherical	2 h		Dose dependent	[101]
Oral Cancer	*Haliclona exigua*	Whole	100–120 nm/flower-like	10 min	KB	0.6 μg/mL	[134]
Ovarian Cancer	*Croton bonplandianum*	Leaf	32 nm/spherical	1 h	PA1	7.5 μg/mL	[125]
	*Scoparia dulcis*	Leaf	150–25 nm/spherical	1 h		Dose dependent	[127]
Pancreatic Cancer	*Dimocarpus longan*	Peel	8–22 nm/spherical	2 h	BxPC 3	38.9 μg/mL	[123]
Prostate Cancer	*Alternanthera sessilis*	Leaf	300–50 nm/spherical	6 h	PC3	6.85 μg/mL	[76]
	*Dimocarpus longan Lour.*	Peel	9–32 nm/cubic	5 h 20 min		Dose dependent	[136]
	*Gracilaria edulis*	Whole	55–99 nm/spherical	-		53.99 μg/mL	[135]
	*Dimocarpus longan*	Peel	8–22 nm/spherical	Overnight	VCaP	87.33 μg/mL	[123]

NP—nanoparticle.

## Supplementary Materials

The following are available online at https://www.mdpi.com/2072-6694/12/4/855/s1, Table S1: Studies AgNPs from Bacteria, Fungi, and Algae that inhibit different cancer cell lines.

## References

[1] Bray F., Ferlay J., Soerjomataram I., Siegel R.L., Torre L.A., Jemal A. (2018). Global cancer statistics 2018: GLOBOCAN estimates of incidence and mortality worldwide for 36 cancers in 185 countries. CA A Cancer J. Clin..

[2] Vickers A. (2004). Alternative cancer cures:“Unproven” or “disproven”?. CA A Cancer J. Clin..

[3] Wang M., Thanou M. (2010). Targeting nanoparticles to cancer. Pharmacol. Res..

[4] Chouhan N. (2018). Silver nanoparticles: Synthesis, characterization and applications. Silver Nanoparticles-Fabrication, Characterization and Applications.

[5] Nguyen K.T. (2011). Targeted nanoparticles for cancer therapy: Promises and challenge. Nanomed. Nanotechnol..

[6] Li X., Cui R., Liu W., Sun L., Yu B., Fan Y., Feng Q., Cui F., Watari F. (2013). The use of nanoscaled fibers or tubes to improve biocompatibility and bioactivity of biomedical materials. J. Nanomater..

[7] Russell A., Hugo W. (1994). 7 antimicrobial activity and action of silver. Prog. Med. Chem..

[8] Lee S.H., Jun B.-H. (2019). Silver nanoparticles: Synthesis and application for nanomedicine. Int. J. Mol. Sci..

[9] Chen X., Schluesener H.J. (2008). Nanosilver: A nanoproduct in medical application. Toxicol. Lett..

[10] Guilger-Casagrande M., de Lima R. (2019). Synthesis of silver nanoparticles mediated by fungi: A Review. Front. Bioeng. Biotechnol..

[11] Ratika K., Vedpriya A. (2013). Biosynthesis and characterization of silver nanoparticles from aqueous leaf extracts of *Carica papaya* and its antibacterial activity. Int. J. Nanomater. Biostructures.

[12] Paulkumar K., Gnanajobitha G., Vanaja M., Rajeshkumar S., Malarkodi C., Pandian K., Annadurai G. (2014). Piper nigrum leaf and stem assisted green synthesis of silver nanoparticles and evaluation of its antibacterial activity against agricultural plant pathogens. Sci. World J..

[13] Nair B., Pradeep T. (2002). Coalescence of nanoclusters and formation of submicron crystallites assisted by *Lactobacillus strains*. Cryst. Growth Des..

[14] Makarov V., Love A., Sinitsyna O., Makarova S., Yaminsky I., Taliansky M., Kalinina N. (2014). “Green” nanotechnologies: Synthesis of metal nanoparticles using plants. Acta Nat..

[15] Fayaz A.M., Balaji K., Kalaichelvan P., Venkatesan R. (2009). Fungal based synthesis of silver nanoparticles—An effect of temperature on the size of particles. Colloids Surf. B Biointerfaces.

[16] Dhanalakshmi P., Azeez R., Rekha R., Poonkodi S., Nallamuthu T. (2012). Synthesis of silver nanoparticles using green and brown seaweeds. Phykos.

[17] Shiny P., Mukherjee A., Chandrasekaran N. (2013). Marine algae mediated synthesis of the silver nanoparticles and its antibacterial efficiency. Int. J. Pharm. Pharm. Sci.

[18] Mie R., Samsudin M.W., Din L.B., Ahmad A., Ibrahim N., Adnan S.N.A. (2014). Synthesis of silver nanoparticles with antibacterial activity using the lichen *Parmotrema praesorediosum*. Int. J. Nanomed..

[19] Zhang X.-F., Liu Z.-G., Shen W., Gurunathan S. (2016). Silver nanoparticles: Synthesis, characterization, properties, applications, and therapeutic approaches. Int. J. Mol. Sci..

[20] Hussain I., Singh N., Singh A., Singh H., Singh S. (2016). Green synthesis of nanoparticles and its potential application. Biotechnol. Lett..

[21] Yoon K.-Y., Byeon J.H., Park J.-H., Hwang J. (2007). Susceptibility constants of *Escherichia coli* and *Bacillus subtilis* to silver and copper nanoparticles. Sci. Total Environ..

[22] Aziz N., Sherwani A., Faraz M., Fatma T., Prasad R. (2019). Illuminating the anticancerous efficacy of a new fungal chassis for silver nanoparticle synthesis. Front. Chem..

[23] Sukirtha R., Priyanka K.M., Antony J.J., Kamalakkannan S., Thangam R., Gunasekaran P., Krishnan M., Achiraman S. (2012). Cytotoxic effect of green synthesized silver nanoparticles using *Melia azedarach* against in vitro HeLa cell lines and lymphoma mice model. Process Biochem..

[24] Boca-Farcau S., Potara M., Simon T., Juhem A., Baldeck P., Astilean S. (2014). Folic acid-conjugated, SERS-labeled silver nanotriangles for multimodal detection and targeted photothermal treatment on human ovarian cancer cells. Mol. Pharm..

[25] Vasanth K., Ilango K., MohanKumar R., Agrawal A., Dubey G.P. (2014). Anticancer activity of *Moringa oleifera* mediated silver nanoparticles on human cervical carcinoma cells by apoptosis induction. Colloids Surf. B Biointerfaces.

[26] Kwon T., Woo H.J., Kim Y.H., Lee H.J., Park K.H., Park S., Youn B. (2012). Optimizing hemocompatibility of surfactant-coated silver nanoparticles in human erythrocytes. J. Nanosci. Nanotechnol..

[27] Asharani P., Hande M.P., Valiyaveettil S. (2009). Anti-proliferative activity of silver nanoparticles. BMC Cell Biol..

[28] Wiley B., Sun Y., Mayers B., Xia Y. (2005). Shape-controlled synthesis of metal nanostructures: The case of silver. Chem. A Eur. J..

[29] Yang Y., Matsubara S., Xiong L., Hayakawa T., Nogami M. (2007). Solvothermal synthesis of multiple shapes of silver nanoparticles and their SERS properties. J. Phys. Chem. C.

[30] Narayanan K.B., Sakthivel N. (2013). Biosynthesis of silver nanoparticles by phytopathogen *Xanthomonas oryzae* pv. oryzae strain BXO8. J. Microbiol. Biotechnol..

[31] Klaus T., Joerger R., Olsson E., Granqvist C.-G. (1999). Silver-based crystalline nanoparticles, microbially fabricated. Proc. Natl. Acad. Sci. USA.

[32] Ingle A., Gade A., Pierrat S., Sonnichsen C., Rai M. (2008). Mycosynthesis of silver nanoparticles using the fungus *Fusarium acuminatum* and its activity against some human pathogenic bacteria. Curr. Nanosci..

[33] Chen S., Carroll D.L. (2004). Silver nanoplates: Size control in two dimensions and formation mechanisms. J. Phys. Chem. B.

[34] Santhoshkumar T., Rahuman A.A., Rajakumar G., Marimuthu S., Bagavan A., Jayaseelan C., Zahir A.A., Elango G., Kamaraj C. (2011). Synthesis of silver nanoparticles using *Nelumbo nucifera* leaf extract and its larvicidal activity against malaria and filariasis vectors. Parasitol. Res..

[35] Vilchis-Nestor A.R., Sánchez-Mendieta V., Camacho-López M.A., Gómez-Espinosa R.M., Camacho-López M.A., Arenas-Alatorre J.A. (2008). Solventless synthesis and optical properties of Au and Ag nanoparticles using *Camellia sinensis* extract. Mater. Lett..

[36] Kreibig U., Vollmer M. (2013). Optical Properties of Metal Clusters.

[37] Kelly K.L., Coronado E., Zhao L.L., Schatz G.C. (2003). The Optical Properties of Metal Nanoparticles: The Influence of Size, Shape, and Dielectric Environment.

[38] Evanoff D.D., Chumanov G. (2004). Size-controlled synthesis of nanoparticles. 2. Measurement of extinction, scattering, and absorption cross sections. J. Phys. Chem. B.

[39] Kajani A.A., Zarkesh-Esfahani S.H., Bordbar A.-K., Khosropour A.R., Razmjou A., Kardi M. (2016). Anticancer effects of silver nanoparticles encapsulated by *Taxus baccata* extracts. J. Mol. Liq..

[40] Kajani A.A., Bordbar A.-K., Esfahani S.H.Z., Khosropour A.R., Razmjou A. (2014). Green synthesis of anisotropic silver nanoparticles with potent anticancer activity using *Taxus baccata* extract. RSC Adv..

[41] Lin L., Qiu P., Cao X., Jin L. (2008). Colloidal silver nanoparticles modified electrode and its application to the electroanalysis of Cytochrome *c*. Electrochim. Acta.

[42] Sankar R., Karthik A., Prabu A., Karthik S., Shivashangari K.S., Ravikumar V. (2013). Origanum vulgare mediated biosynthesis of silver nanoparticles for its antibacterial and anticancer activity. Colloids Surf. B Biointerfaces.

[43] Sepeur S. (2008). Nanotechnology: Technical Basics and Applications.

[44] Meyers M.A., Mishra A., Benson D.J. (2006). Mechanical properties of nanocrystalline materials. Prog. Mater. Sci..

[45] Thakkar K.N., Mhatre S.S., Parikh R.Y. (2010). Biological synthesis of metallic nanoparticles. Nanomed. Nanotechnol. Biol. Med..

[46] Mukherjee P., Ahmad A., Mandal D., Senapati S., Sainkar S.R., Khan M.I., Parishcha R., Ajaykumar P., Alam M., Kumar R. (2001). Fungus-mediated synthesis of silver nanoparticles and their immobilization in the mycelial matrix: A novel biological approach to nanoparticle synthesis. Nano Lett..

[47] Mittal A.K., Chisti Y., Banerjee U.C. (2013). Synthesis of metallic nanoparticles using plant extracts. Biotechnol. Adv..

[48] Kalishwaralal K., Deepak V., Ramkumarpandian S., Nellaiah H., Sangiliyandi G. (2008). Extracellular biosynthesis of silver nanoparticles by the culture supernatant of *Bacillus licheniformis*. Mater. Lett..

[49] Sintubin L., Verstraete W., Boon N. (2012). Biologically produced nanosilver: Current state and future perspectives. Biotechnol. Bioeng..

[50] Prabhu S., Poulose E.K. (2012). Silver nanoparticles: Mechanism of antimicrobial action, synthesis, medical applications, and toxicity effects. Int. Nano Lett..

[51] Karthik L., Kumar G., Kirthi A.V., Rahuman A., Rao K.B. (2014). Streptomyces sp. LK3 mediated synthesis of silver nanoparticles and its biomedical application. Bioprocess Biosyst. Eng..

[52] Vaidyanathan R., Gopalram S., Kalishwaralal K., Deepak V., Pandian S.R.K., Gurunathan S. (2010). Enhanced silver nanoparticle synthesis by optimization of nitrate reductase activity. Colloids Surf. B Biointerfaces.

[53] Kalimuthu K., Babu R.S., Venkataraman D., Bilal M., Gurunathan S. (2008). Biosynthesis of silver nanocrystals by *Bacillus licheniformis*. Colloids Surf. B Biointerfaces.

[54] Golinska P., Wypij M., Ingle A.P., Gupta I., Dahm H., Rai M. (2014). Biogenic synthesis of metal nanoparticles from actinomycetes: Biomedical applications and cytotoxicity. Appl. Microbiol. Biotechnol..

[55] Van Hullebusch E.D., Zandvoort M.H., Lens P.N. (2003). Metal immobilisation by biofilms: Mechanisms and analytical tools. Rev. Environ. Sci. Biotechnol..

[56] Lin Z., Zhou C., Wu J., Zhou J., Wang L. (2005). A further insight into the mechanism of Ag+ biosorption by *Lactobacillus* sp. strain A09. Spectrochim. Acta Part A Mol. Biomol. Spectrosc..

[57] Sintubin L., De Windt W., Dick J., Mast J., van der Ha D., Verstraete W., Boon N. (2009). Lactic acid bacteria as reducing and capping agent for the fast and efficient production of silver nanoparticles. Appl. Microbiol. Biotechnol..

[58] Mohanpuria P., Rana N.K., Yadav S.K. (2008). Biosynthesis of nanoparticles: Technological concepts and future applications. J. Nanoparticle Res..

[59] Dhillon G.S., Brar S.K., Kaur S., Verma M. (2012). Green approach for nanoparticle biosynthesis by fungi: Current trends and applications. Crit. Rev. Biotechnol..

[60] Jain N., Bhargava A., Majumdar S., Tarafdar J., Panwar J. (2011). Extracellular biosynthesis and characterization of silver nanoparticles using *Aspergillus flavus* NJP08: A mechanism perspective. Nanoscale.

[61] Kuppusamy P., Ichwan S.J., Al-Zikri P.N.H., Suriyah W.H., Soundharrajan I., Govindan N., Maniam G.P., Yusoff M.M. (2016). In vitro anticancer activity of Au, Ag nanoparticles synthesized using *Commelina nudiflora* L. aqueous extract against HCT-116 colon cancer cells. Biol. Trace Elem. Res..

[62] Ramasamy M., Lee J.-H., Lee J. (2017). Direct one-pot synthesis of cinnamaldehyde immobilized on gold nanoparticles and their antibiofilm properties. Colloids Surf. B Biointerfaces.

[63] Kumar V., Yadav S.K. (2009). Plant-mediated synthesis of silver and gold nanoparticles and their applications. J. Chem. Technol. Biotechnol..

[64] Mukunthan K., Balaji S. (2012). Cashew apple juice (*Anacardium occidentale* L.) speeds up the synthesis of silver nanoparticles. Int. J. Green Nanotechnol..

[65] Sathishkumar P., Vennila K., Jayakumar R., Yusoff A.R.M., Hadibarata T., Palvannan T. (2016). Phyto-synthesis of silver nanoparticles using *Alternanthera tenella* leaf extract: An effective inhibitor for the migration of human breast adenocarcinoma (MCF-7) cells. Bioprocess Biosyst. Eng..

[66] Vijayaraghavan K., Nalini S.K., Prakash N.U., Madhankumar D. (2012). Biomimetic synthesis of silver nanoparticles by aqueous extract of *Syzygium aromaticum*. Mater. Lett..

[67] Jasuja N.D., Gupta D.K., Reza M., Joshi S.C. (2014). Green Synthesis of AgNPs Stabilized with biowaste and their antimicrobial activities. Braz. J. Microbiol..

[68] Mariselvam R., Ranjitsingh A., Nanthini A.U.R., Kalirajan K., Padmalatha C., Selvakumar P.M. (2014). Green synthesis of silver nanoparticles from the extract of the inflorescence of *Cocos nucifera* (Family: Arecaceae) for enhanced antibacterial activity. Spectrochim. Acta Part A Mol. Biomol. Spectrosc..

[69] Shankar S.S., Rai A., Ahmad A., Sastry M. (2005). Controlling the optical properties of lemongrass extract synthesized gold nanotriangles and potential application in infrared-absorbing optical coatings. Chem. Mater..

[70] Ramteke C., Chakrabarti T., Sarangi B.K., Pandey R.-A. (2012). Synthesis of silver nanoparticles from the aqueous extract of leaves of *Ocimum sanctum* for enhanced antibacterial activity. J. Chem..

[71] Arokiyaraj S., Arasu M.V., Vincent S., Prakash N.U., Choi S.H., Oh Y.-K., Choi K.C., Kim K.H. (2014). Rapid green synthesis of silver nanoparticles from *Chrysanthemum indicum* L and its antibacterial and cytotoxic effects: An in vitro study. Int. J. Nanomed..

[72] Muniyappan N., Nagarajan N. (2014). Green synthesis of silver nanoparticles with *Dalbergia spinosa* leaves and their applications in biological and catalytic activities. Process Biochem..

[73] Vijayaraghavan K., Nalini S.K., Prakash N.U., Madhankumar D. (2012). One step green synthesis of silver nano/microparticles using extracts of *Trachyspermum ammi* and *Papaver somniferum*. Colloids Surf. B Biointerfaces.

[74] Dubey M., Bhadauria S., Kushwah B. (2009). Green synthesis of nanosilver particles from extract of *Eucalyptus hybrida* (safeda) leaf. Dig. J. Nanomater. Biostructures.

[75] Sre P.R., Reka M., Poovazhagi R., Kumar M.A., Murugesan K. (2015). Antibacterial and cytotoxic effect of biologically synthesized silver nanoparticles using aqueous root extract of *Erythrina indica* lam. Spectrochim. Acta Part A Mol. Biomol. Spectrosc..

[76] Firdhouse M.J., Lalitha P. (2013). Biosynthesis of silver nanoparticles using the extract of *Alternanthera sessilis*—Antiproliferative effect against prostate cancer cells. Cancer Nanotechnol..

[77] Flores J.C., Torres V., Popa M., Crespo D., Calderón-Moreno J.M. (2008). Variations in morphologies of silver nanoshells on silica spheres. Colloids Surf. A Physicochem. Eng. Asp..

[78] Jiang J., Oberdörster G., Biswas P. (2009). Characterization of size, surface charge, and agglomeration state of nanoparticle dispersions for toxicological studies. J. Nanoparticle Res..

[79] Shenashen M.A., El-Safty S.A., Elshehy E.A. (2014). Synthesis, morphological control, and properties of silver nanoparticles in potential applications. Part. Part. Syst. Charact..

[80] Schaffer B., Hohenester U., Trügler A., Hofer F. (2009). High-resolution surface plasmon imaging of gold nanoparticles by energy-filtered transmission electron microscopy. Phys. Rev. B.

[81] Song J.Y., Kim B.S. (2009). Rapid biological synthesis of silver nanoparticles using plant leaf extracts. Bioprocess Biosyst. Eng..

[82] Eppler A.S., Rupprechter G., Anderson E.A., Somorjai G.A. (2000). Thermal and chemical stability and adhesion strength of Pt nanoparticle arrays supported on silica studied by transmission electron microscopy and atomic force microscopy. J. Phys. Chem. B.

[83] Chithrani B.D., Ghazani A.A., Chan W.C. (2006). Determining the size and shape dependence of gold nanoparticle uptake into mammalian cells. Nano Lett..

[84] Gnanadhas D.P., Thomas M.B., Thomas R., Raichur A.M., Chakravortty D. (2013). Interaction of silver nanoparticles with serum proteins affects their antimicrobial activity in vivo. Antimicrob. Agents Chemother..

[85] Kathiravan V., Ravi S., Ashokkumar S. (2014). Synthesis of silver nanoparticles from *Melia dubia* leaf extract and their in vitro anticancer activity. Spectrochim. Acta Part A Mol. Biomol. Spectrosc..

[86] Sun S., Murray C.B., Weller D., Folks L., Moser A. (2000). Monodisperse FePt nanoparticles and ferromagnetic FePt nanocrystal superlattices. Science.

[87] Strasser P., Koh S., Anniyev T., Greeley J., More K., Yu C., Liu Z., Kaya S., Nordlund D., Ogasawara H. (2010). Lattice-strain control of the activity in dealloyed core–shell fuel cell catalysts. Nat. Chem..

[88] Baharara J., Namvar F., Ramezani T., Mousavi M., Mohamad R. (2015). Silver nanoparticles biosynthesized using *Achillea biebersteinii* flower extract: Apoptosis induction in MCF-7 cells via caspase activation and regulation of Bax and Bcl-2 gene expression. Molecules.

[89] Castro-Aceituno V., Ahn S., Simu S.Y., Singh P., Mathiyalagan R., Lee H.A., Yang D.C. (2016). Anticancer activity of silver nanoparticles from *Panax ginseng* fresh leaves in human cancer cells. Biomed. Pharmacother..

[90] Devaraj P., Aarti C., Kumari P. (2014). Synthesis and characterization of silver nanoparticles using *Tabernae montana* divaricata and its cytotoxic activity against MCF7 cell line. Int. J. Pharm. Pharm. Sci..

[91] Elangovan K., Elumalai D., Anupriya S., Shenbhagaraman R., Kaleena P., Murugesan K. (2015). Phyto mediated biogenic synthesis of silver nanoparticles using leaf extract of *Andrographis echioides* and its bio-efficacy on anticancer and antibacterial activities. J. Photochem. Photobiol. B Biol..

[92] Heydari R., Rashidipour M. (2015). Green synthesis of silver nanoparticles using extract of oak fruit hull (Jaft): Synthesis and in vitro cytotoxic effect on MCF-7 cells. Int. J. Breast Cancer.

[93] Jeyaraj M., Sathishkumar G., Sivanandhan G., MubarakAli D., Rajesh M., Arun R., Kapildev G., Manickavasagam M., Thajuddin N., Premkumar K. (2013). Biogenic silver nanoparticles for cancer treatment: An experimental report. Colloids Surf. B Biointerfaces.

[94] Lalitha P. (2015). Apoptotic efficacy of biogenic silver nanoparticles on human breast cancer MCF-7 cell lines. Prog. Biomater..

[95] Mittal A.K., Thanki K., Jain S., Banerjee U.C. (2016). Comparative studies of anticancer and antimicrobial potential of bioinspired silver and silver-selenium nanoparticles. Appl. Nanomed..

[96] Mukherjee S., Chowdhury D., Kotcherlakota R., Patra S. (2014). Potential theranostics application of bio-synthesized silver nanoparticles (4-in-1 system). Theranostics.

[97] Patra S., Mukherjee S., Barui A.K., Ganguly A., Sreedhar B., Patra C.R. (2015). Green synthesis, characterization of gold and silver nanoparticles and their potential application for cancer therapeutics. Mater. Sci. Eng. C.

[98] Ramar M., Manikandan B., Marimuthu P.N., Raman T., Mahalingam A., Subramanian P., Karthick S., Munusamy A. (2015). Synthesis of silver nanoparticles using *Solanum trilobatum* fruits extract and its antibacterial, cytotoxic activity against human breast cancer cell line MCF 7. Spectrochim. Acta Part A Mol. Biomol. Spectrosc..

[99] Reddy N.J., Vali D.N., Rani M., Rani S.S. (2014). Evaluation of antioxidant, antibacterial and cytotoxic effects of green synthesized silver nanoparticles by *Piper longum* fruit. Mater. Sci. Eng. C.

[100] Sathishkumar G., Gobinath C., Wilson A., Sivaramakrishnan S. (2014). Dendrophthoe falcata (Lf) Ettingsh (*Neem mistletoe*): A potent bioresource to fabricate silver nanoparticles for anticancer effect against human breast cancer cells (MCF-7). Spectrochim. Acta Part A Mol. Biomol. Spectrosc..

[101] Sathishkumar P., Preethi J., Vijayan R., Yusoff A.R.M., Ameen F., Suresh S., Balagurunathan R., Palvannan T. (2016). Anti-acne, anti-dandruff and anti-breast cancer efficacy of green synthesised silver nanoparticles using Coriandrum sativum leaf extract. J. Photochem. Photobiol. B Biol..

[102] Sharma D., Ledwani L., Bhatnagar N. (2015). Antimicrobial and cytotoxic potential of silver nanoparticles synthesized using *Rheum emodi* roots extract. Ann. West Univ. Timis. Ser. Chem..

[103] Shawkey A.M., Rabeh M.A., Abdulall A.K., Abdellatif A.O. (2013). Green nanotechnology: Anticancer activity of silver nanoparticles using *Citrullus colocynthis* aqueous extracts. Adv. Life Sci. Technol..

[104] Venugopal K., Rather H., Rajagopal K., Shanthi M., Sheriff K., Illiyas M., Rather R., Manikandan E., Uvarajan S., Bhaskar M. (2017). Synthesis of silver nanoparticles (Ag NPs) for anticancer activities (MCF 7 breast and A549 lung cell lines) of the crude extract of *Syzygium aromaticum*. J. Photochem. Photobiol. B Biol..

[105] Devi J., Bhimba B. (2012). Anticancer activity of silver nanoparticles synthesized by the seaweed *Ulva lactuca in vitro*. Open Access Sci. Rep..

[106] Mishra A., Mehdi S.J., Irshad M., Ali A., Sardar M., Moshahid M., Rizvi A. (2012). Effect of biologically synthesized silver nanoparticles on human cancer cells. Sci. Adv. Mater..

[107] Nakkala J.R., Mata R., Gupta A.K., Sadras S.R. (2014). Biological activities of green silver nanoparticles synthesized with *Acorous calamus* rhizome extract. Eur. J. Med. Chem..

[108] Rajkuberan C., Sudha K., Sathishkumar G., Sivaramakrishnan S. (2015). Antibacterial and cytotoxic potential of silver nanoparticles synthesized using latex of *Calotropis gigantea* L.. Spectrochim. Acta Part A Mol. Biomol. Spectrosc..

[109] Chanthini A.B., Balasubramani G., Ramkumar R., Sowmiya R., Balakumaran M.D., Kalaichelvan P.T., Perumal P. (2015). Structural characterization, antioxidant and in vitro cytotoxic properties of seagrass, *Cymodocea serrulata* (R. Br.) Asch. & Magnus mediated silver nanoparticles. J. Photochem. Photobiol. B Biol..

[110] Vijistella Bai G. (2014). Green synthesis of silver nanostructures against human cancer cell lines and certain pathogens. Int. J. Pharm. Chem. Biol. Sci..

[111] Jeyaraj M., Rajesh M., Arun R., MubarakAli D., Sathishkumar G., Sivanandhan G., Dev G.K., Manickavasagam M., Premkumar K., Thajuddin N. (2013). An investigation on the cytotoxicity and caspase-mediated apoptotic effect of biologically synthesized silver nanoparticles using *Podophyllum hexandrum* on human cervical carcinoma cells. Colloids Surf. B Biointerfaces.

[112] Govindaraju K., Krishnamoorthy K., Alsagaby S.A., Singaravelu G., Premanathan M. (2015). Green synthesis of silver nanoparticles for selective toxicity towards cancer cells. IET Nanobiotechnology.

[113] Mata R., Nakkala J.R., Sadras S.R. (2015). Catalytic and biological activities of green silver nanoparticles synthesized from *Plumeria alba* (frangipani) flower extract. Mater. Sci. Eng. C.

[114] Manikandan R., Manikandan B., Raman T., Arunagirinathan K., Prabhu N.M., Basu M.J., Perumal M., Palanisamy S., Munusamy A. (2015). Biosynthesis of silver nanoparticles using ethanolic petals extract of *Rosa indica* and characterization of its antibacterial, anticancer and anti-inflammatory activities. Spectrochim. Acta Part A Mol. Biomol. Spectrosc..

[115] Prabhu D., Arulvasu C., Babu G., Manikandan R., Srinivasan P. (2013). Biologically synthesized green silver nanoparticles from leaf extract of *Vitex negundo* L. induce growth-inhibitory effect on human colon cancer cell line HCT15. Process Biochem..

[116] Arunachalam K.D., Arun L.B., Annamalai S.K., Arunachalam A.M. (2015). Potential anticancer properties of bioactive compounds of *Gymnema sylvestre* and its biofunctionalized silver nanoparticles. Int. J. Nanomed..

[117] Nayak D., Pradhan S., Ashe S., Rauta P.R., Nayak B. (2015). Biologically synthesised silver nanoparticles from three diverse family of plant extracts and their anticancer activity against epidermoid A431 carcinoma. J. Colloid Interface Sci..

[118] Salehi S., Shandiz S.A.S., Ghanbar F., Darvish M.R., Ardestani M.S., Mirzaie A., Jafari M. (2016). Phytosynthesis of silver nanoparticles using *Artemisia marschalliana* Sprengel aerial part extract and assessment of their antioxidant, anticancer, and antibacterial properties. Int. J. Nanomed..

[119] Pandian A.M.K., Karthikeyan C., Rajasimman M., Dinesh M. (2015). Synthesis of silver nanoparticle and its application. Ecotoxicol. Environ. Saf..

[120] Kumar B., Smita K., Seqqat R., Benalcazar K., Grijalva M., Cumbal L. (2016). In vitro evaluation of silver nanoparticles cytotoxicity on Hepatic cancer (Hep-G2) cell line and their antioxidant activity: Green approach for fabrication and application. J. Photochem. Photobiol. B Biol..

[121] Satyavani K., Gurudeeban S., Ramanathan T., Balasubramanian T. (2011). Biomedical potential of silver nanoparticles synthesized from calli cells of *Citrullus colocynthis* (L.) Schrad. J. Nanobiotechnology.

[122] Satyavani K., Gurudeeban S., Ramanathan T., Balasubramanian T. (2012). Toxicity study of silver nanoparticles synthesized from *Suaeda monoica* on Hep-2 cell line. Avicenna J. Med Biotechnol..

[123] He Y., Du Z., Ma S., Liu Y., Li D., Huang H., Jiang S., Cheng S., Wu W., Zhang K. (2016). Effects of green-synthesized silver nanoparticles on lung cancer cells in vitro and grown as xenograft tumors in vivo. Int. J. Nanomed..

[124] Xia Q.H., Ma Y.J., Wang J.W. (2016). Biosynthesis of silver nanoparticles using *Taxus yunnanensis* callus and their antibacterial activity and cytotoxicity in human cancer cells. Nanomaterials.

[125] Gurunathan S., Jeong J.-K., Han J.W., Zhang X.-F., Park J.H., Kim J.-H. (2015). Multidimensional effects of biologically synthesized silver nanoparticles in *Helicobacter pylori*, *Helicobacter felis*, and human lung (L_132_) and lung carcinoma A_549_ cells. Nanoscale Res. Lett..

[126] Kanipandian N., Thirumurugan R. (2014). A feasible approach to phyto-mediated synthesis of silver nanoparticles using industrial crop *Gossypium hirsutum* (cotton) extract as stabilizing agent and assessment of its in vitro biomedical potential. Ind. Crop. Prod..

[127] Khanra K., Panja S., Choudhuri I., Chakraborty A., Bhattacharyya N. (2015). Evaluation of antibacterial activity and cytotoxicity of green synthesized silver nanoparticles using *Scoparia dulcis*. Nano Biomed Eng.

[128] Khanra K., Panja S., Choudhuri I., Chakraborty A., Bhattacharyya N. (2016). Antimicrobial and cytotoxicity effect of silver nanoparticle synthesized by *Croton bonplandianum* Baill. leaves. Nanomed. J..

[129] Majeed S., bin Abdullah M.S., Dash G.K., Ansari M.T., Nanda A. (2016). Biochemical synthesis of silver nanoprticles using filamentous fungi *Penicillium decumbens* (MTCC-2494) and its efficacy against A-549 lung cancer cell line. Chin. J. Nat. Med..

[130] Palaniappan P., Sathishkumar G., Sankar R. (2015). Fabrication of nano-silver particles using *Cymodocea serrulata* and its cytotoxicity effect against human lung cancer A549 cells line. Spectrochim. Acta Part A Mol. Biomol. Spectrosc..

[131] Venkatesan B., Subramanian V., Tumala A., Vellaichamy E. (2014). Rapid synthesis of biocompatible silver nanoparticles using aqueous extract of *Rosa damascena* petals and evaluation of their anticancer activity. Asian Pac. J. Trop. Med..

[132] Mollick M.M.R., Rana D., Dash S.K., Chattopadhyay S., Bhowmick B., Maity D., Mondal D., Pattanayak S., Roy S., Chakraborty M. (2015). Studies on green synthesized silver nanoparticles using *Abelmoschus esculentus* (L.) pulp extract having anticancer (in vitro) and antimicrobial applications. Arab. J. Chem..

[133] Banerjee K., Das S., Choudhury P., Ghosh S., Baral R., Choudhuri S.K. (2017). A novel approach of synthesizing and evaluating the anticancer potential of silver oxide nanoparticles in vitro. Chemotherapy.

[134] Inbakandan D., Kumar C., Bavanilatha M., Ravindra D.N., Kirubagaran R., Khan S.A. (2016). Ultrasonic-assisted green synthesis of flower like silver nanocolloids using marine sponge extract and its effect on oral biofilm bacteria and oral cancer cell lines. Microb. Pathog..

[135] Priyadharshini R.I., Prasannaraj G., Geetha N., Venkatachalam P. (2014). Microwave-mediated extracellular synthesis of metallic silver and zinc oxide nanoparticles using macro-algae (Gracilaria edulis) extracts and its anticancer activity against human PC3 cell lines. Appl. Biochem. Biotechnol..

[136] He Y., Du Z., Ma S., Cheng S., Jiang S., Liu Y., Li D., Huang H., Zhang K., Zheng X. (2016). Biosynthesis, antibacterial activity and anticancer effects against prostate cancer (PC-3) cells of silver nanoparticles using Dimocarpus Longan Lour. Nanoscale Res. Lett..

[137] Gliga A.R., Skoglund S., Wallinder I.O., Fadeel B., Karlsson H.L. (2014). Size-dependent cytotoxicity of silver nanoparticles in human lung cells: The role of cellular uptake, agglomeration and Ag release. Part. Fibre Toxicol..

[138] Priyaragini S., Sathishkumar S., Bhaskararao K. (2013). Biosynthesis of silver nanoparticles using actinobacteria and evaluating its antimicrobial and cytotoxicity activity. Int. J. Pharm. Pharm. Sci..

[139] Kim J.-H., Lee Y., Kim E.-J., Gu S., Sohn E.J., Seo Y.S., An H.J., Chang Y.-S. (2014). Exposure of iron nanoparticles to *Arabidopsis thaliana* enhances root elongation by triggering cell wall loosening. Environ. Sci. Technol..

[140] Birbrair A., Zhang T., Wang Z.-M., Messi M.L., Olson J.D., Mintz A., Delbono O. (2014). Type-2 pericytes participate in normal and tumoral angiogenesis. Am. J. Physiol. Cell Physiol..

[141] Bhat T.A., Singh R.P. (2008). Tumor angiogenesis—A potential target in cancer chemoprevention. Food Chem. Toxicol..

[142] Folkman J. (2002). Role of angiogenesis in tumor growth and metastasis. Seminars in Oncology.

[143] Shen H.-H., Chan E.C., Lee J.H., Bee Y.-S., Lin T.-W., Dusting G.J., Liu G.-S. (2015). Nanocarriers for treatment of ocular neovascularization in the back of the eye: New vehicles for ophthalmic drug delivery. Nanomedicine.

[144] Khandia R., Munjal A., Bangrey R., Mehra R., Dhama K., Sharma N. (2015). Evaluation of silver nanoparticle mediated reduction of neovascularisation (angiogenesis) in chicken model. Adv. Anim. Vet. Sci.

[145] Collins K., Jacks T., Pavletich N.P. (1997). The cell cycle and cancer. Proc. Natl. Acad. Sci. USA.

[146] Chang Y.-J., Tai C.-J., Kuo L.-J., Wei P.-L., Liang H.-H., Liu T.-Z., Wang W., Tai C.-J., Ho Y.-S., Wu C.-H. (2011). Glucose-regulated protein 78 (GRP78) mediated the efficacy to curcumin treatment on hepatocellular carcinoma. Ann. Surg. Oncol..

[147] Mao X., Seidlitz E., Truant R., Hitt M., Ghosh H.P. (2004). Re-expression of TSLC1 in a non-small-cell lung cancer cell line induces apoptosis and inhibits tumor growth. Oncogene.

[148] Bharadwaj Punita S. (2012). Silver or silver nanoparticle a safety or a risk. J. Environ. Res. Dev..

[149] Mahmoudi M., Serpooshan V. (2012). Silver-coated engineered magnetic nanoparticles are promising for the success in the fight against antibacterial resistance threat. ACS Nano.

[150] Roy A., Bulut O., Some S., Mandal A.K., Yilmaz M.D. (2019). Green synthesis of silver nanoparticles: Biomolecule-nanoparticle organizations targeting antimicrobial activity. RSC Adv..

[151] Ahmed K.B.R., Nagy A.M., Brown R.P., Zhang Q., Malghan S.G., Goering P.L. (2017). Silver nanoparticles: Significance of physicochemical properties and assay interference on the interpretation of in vitro cytotoxicity studies. Toxicol. Vitr..

[152] Dziedzic A., Kubina R., Bułdak R.J., Skonieczna M., Cholewa K. (2016). Silver nanoparticles exhibit the dose-dependent anti-proliferative effect against human squamous carcinoma cells attenuated in the presence of berberine. Molecules.

[153] Skonieczna M., Hudy D. (2018). Biological activity of silver nanoparticles and their applications in anticancer therapy. Silver Nanoparticles Fabr. Charact. Appl..

[154] Braydich-Stolle L.K., Lucas B., Schrand A., Murdock R.C., Lee T., Schlager J.J., Hussain S.M., Hofmann M.-C. (2010). Silver nanoparticles disrupt GDNF/Fyn kinase signaling in spermatogonial stem cells. Toxicol. Sci..

[155] Swanner J., Fahrenholtz C.D., Tenvooren I., Bernish B.W., Sears J.J., Hooker A., Furdui C.M., Alli E., Li W., Donati G.L. (2019). Silver nanoparticles selectively treat triple-negative breast cancer cells without affecting non-malignant breast epithelial cells in vitro and in vivo. FASEB Bioadv..

[156] Gurunathan S., Raman J., Malek S.N.A., John P.A., Vikineswary S. (2013). Green synthesis of silver nanoparticles using Ganoderma neo-japonicum Imazeki: A potential cytotoxic agent against breast cancer cells. Int. J. Nanomed..

[157] Madamsetty V.S., Mukherjee A., Mukherjee S. (2019). Recent trends of the bio-inspired nanoparticles in cancer theranostics. Front. Pharmacol..

[158] García-Contreras R., Argueta-Figueroa L., Mejía-Rubalcava C., Jiménez-Martínez R., Cuevas-Guajardo S., Sánchez-Reyna P.A., Mendieta-Zeron H. (2011). Perspectives for the use of silver nanoparticles in dental practice. Int. Dent. J..

[159] Rai M., Ingle A.P., Paralikar P., Gupta I., Medici S., Santos C.A. (2017). Recent advances in use of silver nanoparticles as antimalarial agents. Int. J. Pharm..

[160] Park K., Park E.-J., Chun I.K., Choi K., Lee S.H., Yoon J., Lee B.C. (2011). Bioavailability and toxicokinetics of citrate-coated silver nanoparticles in rats. Arch. Pharmacal Res..

[161] Samberg M.E., Lin Z., Monteiro-Riviere N.A., Bhushan B. (2014). In vitro and in vivo toxicity and pharmacokinetics of silver nanoparticles. Encyclopedia of Nanotechnology.

[162] Ravanshad R., Karimi Zadeh A., Amani A.M., Mousavi S.M., Hashemi S.A., Savar Dashtaki A., Mirzaei E., Zare B. (2018). Application of nanoparticles in cancer detection by Raman scattering based techniques. Nano Rev. Exp..

