# Supplementary material for: Green Chemistry Synthesis of Silver Nanoparticles and Their Potential Anticancer Effects
Source: Cancers (Basel). 2020 Apr 1;12(4):855. doi: 10.3390/cancers12040855 (PMC7226404; doi:10.3390/cancers12040855)
Supplement: Supplementary file 1 [file cancers-12-00855-s001.pdf]

Table S1. Studies AgNPs from Bacterai,Fungi,Algae that inhibit different cancer cell lines.

Cell Line	Size in nm	Biogenic Agent	IC50 Value	References
HL-60 (human promyeloid leukaemia cells)	33.5	*Aspergillus flavus (*Algae*)*	-	[1]
MDA-MB-231 (human breast cancer cells)	-	*Ganoderma neo-japonicum(*Algae*)*	6 μg/ml	[2]
DLA (Dalton's lymphoma ascites)	50	*Bacillus licheniformi(*Bacteria*)*	500nM	[3]
DLA (Dalton's lymphoma ascites)	10-50	*Anabaena doliolum (*Bacteria*)*	20 μg/ml	[4]
HT-29(human colon cancer cell line)	149-397	*Penicillium spp.* (Fungi)	30 μg/ml	[5]
MCF-7 (breast cancer cell line)	75	*Taxus baccata* (Yeast)	0.25 μg/ml	[6]
Hela	-	*Andrographis Paniculata* (Yeast)	59 μg/ml	[7]
MCF-7 (breast cancer cell line)	60-90	*Dodonaea viscose* (Yeast)	98.03 μg/ml	[8]
MCF-7 (breast cancer cell line)	22	*Sesbania grandiflora* (Yeast)	20 μg/ml	[9]

References:

1. Sulaiman, G.M.; Hussien, H.T.; Saleem, M.M.N.M. Biosynthesis of silver nanoparticles synthesized by Aspergillus flavus and their antioxidant, antimicrobial and cytotoxicity properties. *Bull. Mater. Sci.* **2015**, *38*, 639–644.
2. Gurunathan, S.; Raman, J.; Abd Malek, S.N.; John, P.A.; Vikineswary, S. Green synthesis of silver nanoparticles using Ganoderma neo-japonicum Imazeki: a potential cytotoxic agent against breast cancer cells. *Int. J. Nanomed.* **2013**, *8*, 4399–4413.
3. Sriram, M.I.; Kanth, S.B.M.; Kalishwaralal, K.; Gurunathan, S. Antitumor activity of silver nanoparticles in Dalton's lymphoma ascites tumor model. *Int. J. Nanomed*. **2010**, *5*, 753–762.
4. Singh, G.; Babele, P.K.; Shahi, S.K.; Sinha, R.P.; Tyagi, M.B.; Kumar, A. Green synthesis of silver nanoparticles using cell extracts of Anabaena doliolum and screening of its antibacterial and antitumor activity. *J. Microbiol. Biotechnol.* **2014**, *24*, 1354-1367.
5. Verma, S.; Senguttuvan, A.; Velrajan, M. Anticancer and antibacterial activity of silver nanoparticles biosynthesized by Penicillium spp. and its synergistic effect with antibiotics. *J. Microbiol. Biotechnol*. **2013**, *3*, 54–71.
6. Kajani, A.A.; Bordbar, A.-K.; Zarkesh Esfahani, S.H.; Khosropour, A.R.; Razmjou, A. Green synthesis of anisotropic silver nanoparticles with potent anticancer activity using Taxus baccata extract. *RSC Adv.* **2014**. *4*, 61394–61403.
7. Dhamodaran, M.; Kavitha, S. In-Vitro Anticancer Activity of Silver Nanoparticle in Terpenoid for Andrographis Paniculata (Ag-Nps TAP) by MTT Assay Method against Hela &Hep-2. *IJARCS* **2015**, *2*, 8–13.
8. Giridharan, T.; Masi, C.; Sivalingam, S.; Arumugam, P. Studies on green synthesis, characterization and anti-proliferative potential of silver nano particle using Dodonaea viscosa and Capparis decidua. *Biosci. Biotechnol. Res. Asia* **2014**. *11*, 665–673.
9. Jeyaraj, M.; Sathishkumar, G.; Sivanandhan, G.; MubarakAli, D.; Rajesh, M.; Arun, R.; Kapildev, G.; Manickavasagam, M.; Thajuddin, N.; Premkumar, K.; et al. Biogenic silver nanoparticles for cancer treatment: an experimental report. *Colloid Surf. Biointerfac*. **2013**, *106*, 86–92.
